# Supplementary material for: Dual roles for ATP in the regulation of phase separated protein aggregates in Xenopus oocyte nucleoli
Source: eLife. 2018 Jul 17;7:e35224. doi: 10.7554/eLife.35224 (PMC6050040; doi:10.7554/eLife.35224)
Supplement: Table 1—source data 1. [file elife-35224-table1-data1.docx]

**Table 1-source data 1**

**Retained Proteins Following Depletion of Soluble Nuclear Components**

| actb | ftsj3 | narg2 | polr1b | rpl23a | sycp2l |
| --- | --- | --- | --- | --- | --- |
| aldoc | gapdh | nasp | polr1c | rpl31 | sytl2 |
| anp32e | gar1 | nat10 | polr2e | rpl4 | tbl3 |
| apoa1 | gnl3 | ncl | prim1 | rpl6 | top2a |
| atp5a1 | gpc3 | nhp2l1 | prim2 | rpl7 | tpr |
| atp5b | gtfe2h1 | noc2l | prpf19 | rpl7a | tubb4b |
| bop1 | gtf2h2 | nolc1 | prpf6 | rpl8 | u2af2 |
| c16orf88 | gtpbp4 | nop2 | prpf8 | rps13 | uba52 |
| cebpz | heatr2 | nop56 | ptbp1 | rps14 | urb1 |
| coil | hist1h2aa | nop58 | pwp2 | rps16 | vcp |
| cse1l | hist2h3d | npm1 | rfc1 | rpsa | vtga2 |
| csnk2b | hspa8 | npm3 | rfc2 | rrp9 | vtgb1 |
| ddx18 | jup | nrip3 | rfc3 | ruvbl2 | wdhd1 |
| ddx21 | kif15 | nup155 | rfc4 | sec13 | wdr18 |
| dhx15 | lama1 | nup205 | rfc5 | serpina6 | wdr3 |
| dkc1 | ldhb | nup210 | rpgrip1l | set | wdr36 |
| dsp | lmnb3 | nup93 | rpl10a | smarca5 | wrn |
| dus3l | lrrc41 | pak1ip1 | rpl12 | snrnp200 | xab2 |
| eef1a2 | mcm2 | pdcd11 | rpl14 | snrpd3 | xnf7 |
| eftud2 | mcm5 | pelp1 | rpl18 | sri | zdhhc8 |
| eno1 | mcm6 | pes1 | rpl18a | srrm1 |  |
| esf1 | mdh1 | pole | rpl19 | srrm2 |  |
| fbl | mdn1 | polr1a | rpl22 | srsf5 |  |
|  |  |  |  |  |  |

Proteomic analysis of the deposited samples was performed in technical triplicate. Aggregated proteins, labeled “purple wash,” that were identified with >99% confidence were included in GOanalysis**.**
