# Supplementary material for: Dual roles for ATP in the regulation of phase separated protein aggregates in Xenopus oocyte nucleoli
Source: eLife. 2018 Jul 17;7:e35224. doi: 10.7554/eLife.35224 (PMC6050040; doi:10.7554/eLife.35224)
Supplement: Table 1—source data 2. [file elife-35224-table1-data2.docx]

**Table 1-source data 2.** Key with brief descriptions of the data sets.

| Treatment Name | Treatment Applied |
| --- | --- |
| 1. Unknown Blank | Buffer |
| 2. IGS | Carroll Iso and Carroll Buffer Wash |
| 3. OR2 | Carroll Iso and OR2 Buffer Wash |
| 4. Purple Envelope | Manually Isolated Env. from Scalenghe Iso |
| 5. Purple SN | Scalenghe Iso, super from OR2 wash |
| 6. Purple Wash | Scalenghe Iso, aggregates from OR2 wash |
| 7. RNase | Aggregates from Carroll Iso and OR2 + RNase A Wash |
| 8. SN1-1 | Carroll Iso and OR2 Buffer Wash (supernatant) |
| 9. SN6 | Carroll Iso and OR2 Buffer Wash (supernatant) |
| 10. WashN3 | Carroll Iso, OR2 Buffer Washed Aggregates |
| 11. WashN3 | Carroll Iso, OR2 Buffer Washed Aggregates |
| 12. WashN63 | Carroll Iso, OR2 Buffer Washed Aggregates |
